# Complete Genome Sequence of an Isolate of *Passiflora chlorosis virus* from Passion Fruit (*Passiflora edulis* Sims)

**DOI:** 10.3390/plants11141838

**Published:** 2022-07-13

**Authors:** Patricia Fresnillo, Sara Jover-Gil, Alon Samach, Héctor Candela

**Affiliations:** 1 The Robert H. Smith Faculty of Agriculture, Food and Environment, The Robert H. Smith Institute of Plant Sciences and Genetics in Agriculture, The Hebrew University of Jerusalem, Rehovot 76100, Israel; patricia.fresnillo@mail.huji.ac.il (P.F.); alon.samach@mail.huji.ac.il (A.S.); 2 Instituto de Bioingeniería, Universidad Miguel Hernández, Campus de Elche, 03202 Elche, Spain; sjover@umh.es

**Keywords:** passion fruit, *Potyviridae*, potyviruses, *Passiflora edulis*, *Passiflora chlorosis virus*

## Abstract

We report the first complete genome sequence of an isolate of *Passiflora chlorosis virus* (PaCV), a member of the *Potyviridae* family, identified in passion fruit (*Passiflora edulis* Sims) plants grown in Israel. The assembled genome is 9672 nucleotides long and encodes a 3084 amino acids polyprotein that is predicted to be proteolytically cleaved into 10 mature peptides. Our analysis of the genome sequence shows that PaCV is a distinct species, sharing 68.5% nucleotide sequence identity and 71.5% amino acid sequence identity with isolates of the bean common mosaic necrosis virus (BCMNV), the most closely related virus classified within the genus *Potyvirus*. Using quantitative PCR, we detected the virus in RNA samples from leaves exhibiting symptoms of infection, with higher levels in clearly chlorotic leaves, but not in those from healthy leaves.

## 1. Introduction

By allowing rapid and affordable access to their complete genome sequences, next-generation sequencing technologies are greatly facilitating the discovery and characterization of new species of plant viruses. Passion fruit (*Passiflora edulis* Sims) is an important crop that is cultivated in tropical and subtropical areas for its edible fruit, but its yield can be compromised by different pathogens, including a growing number of viruses of the *Potyviridae* family [1,2,3]. One such virus, named passiflora chlorosis virus (PaCV), was first reported in Florida (USA) in 2004, infecting passion fruit plants that exhibited chlorotic symptoms and had cylindrical inclusions in their cells [4,5]. In 2008, PaCV was ratified as a new species of the genus *Potyvirus* by the International Committee of Taxonomy of Viruses (ICTV) [6]. Additional isolates of the same species have been collected in France, infecting the legume *Bituminaria bituminosa* [7], and in Germany [3], also infecting passion fruit. Despite a long time having elapsed since those early reports, only four partial sequences are available in the GenBank database for this species (with accession numbers EU334546, EU334547, FR694185, and DQ860147), all of them limited to parts of the genome encoding the RNA-dependent RNA polymerase NIb and the coat protein. In this article, we report the complete genome of a new PaCV isolate from Israel, which we identified in passion fruit plants that exhibited chlorotic symptoms. To our knowledge, ours is the first complete genome sequence submitted for an isolate of this species.

## 2. Results and Discussion

In the course of a de novo assembly of the transcriptome of ovary tissues of passion fruit plants, performed as described in Materials and Methods, we identified two different complete viral genomes in two RNA samples. Using BLAST, we found that the most abundant genome sequence corresponded to the Rehovot isolate of Passiflora latent virus (PLV; MH379331.1), which we have previously reported in asymptomatic plants from the same location [2]. In these samples, the PLV genome was represented by 31,207 (0.10%) and 43,157 (0.14%) reads, respectively. The second genome sequence corresponded to *Passiflora chlorosis virus* (PaCV) [4], a species for which a complete genome sequence had not been previously reported. This new genome sequence was represented by 5694 (0.02%) and 17,701 (0.06%) reads, respectively, and has been deposited in GenBank with accession number MT263075. 

**Figure 1:**
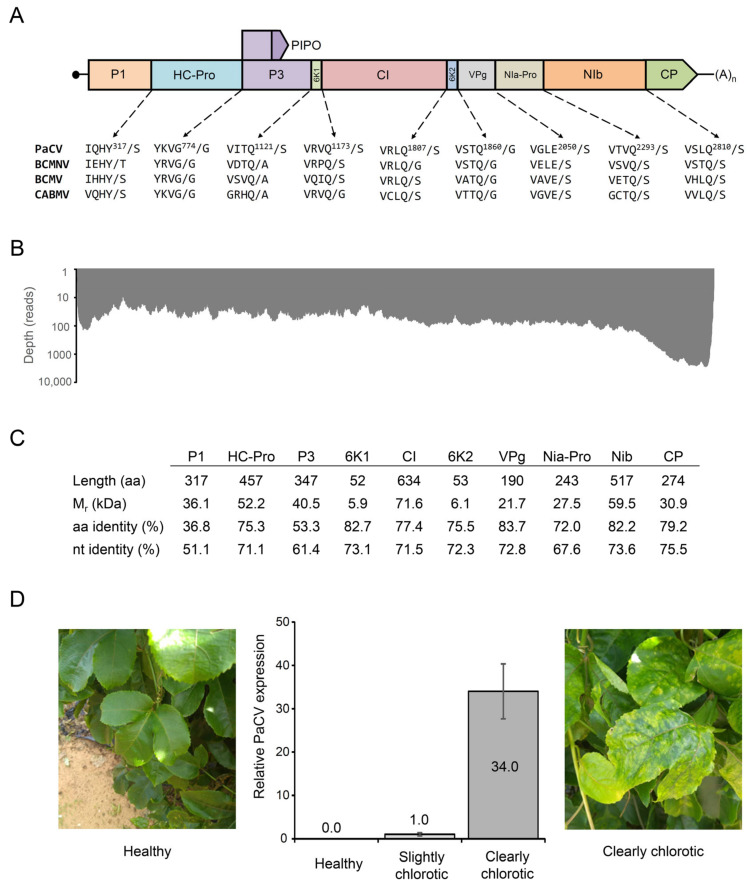
Characteristics of passiflora chlorosis virus (PaCV). (**A**) Genome organization. The structure of the polyprotein is shown. The predicted cleavage sites of the PaCV polyprotein are compared to those of the BCMNV (U19287), BCMV (AJ312437), and CABMV (AF348210) polyproteins. (**B**) Sequencing depth along the genome sequence of PaCV. (**C**) Some characteristics of the ten mature peptides encoded in the genome of PaCV. The sequence identity percentages are based on global alignments of the nucleotide (nt) and amino acid (aa) sequences of PaCV and BCMNV. (**D**) Detection of PaCV in samples from healthy and chlorotic plants using RT-PCR. Error bars indicate the standard deviation (n = 3). Leaves from a healthy passion fruit plant (left) and from a clearly chlorotic plant showing symptoms of PaCV infection (right) are also shown.

**Figure 2:**
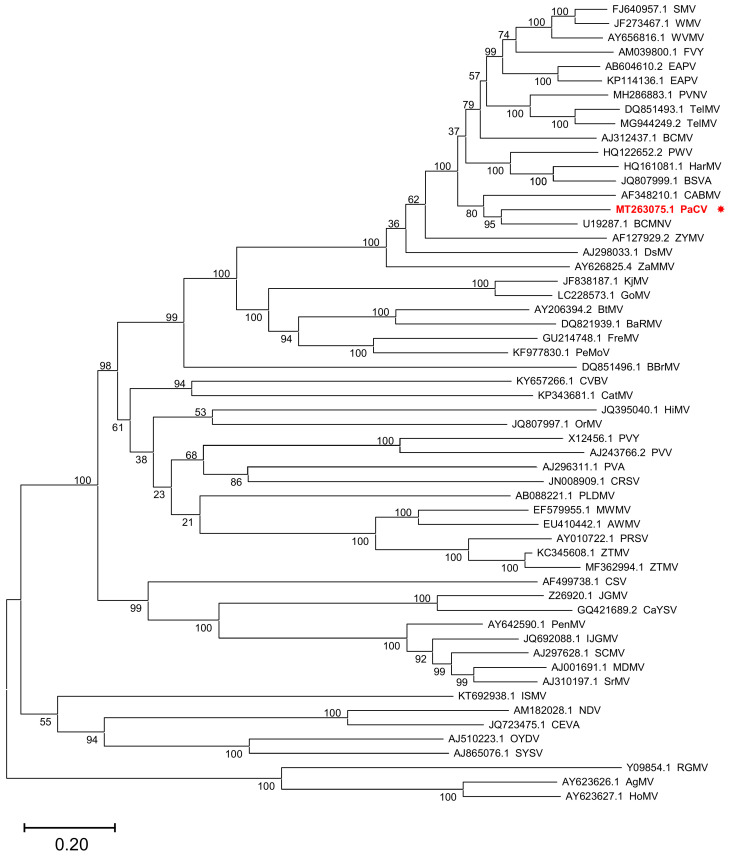
Phylogenetic analysis of passiflora chlorosis virus. The evolutionary history was inferred by using the maximum likelihood method based on the Le Gascuel 2008 model (LG + G + I + F) model [23]. The tree with the highest log likelihood (−184,964.05) is shown. The percentage of trees in which the sequences clustered together is shown next to the branches. Initial trees for the heuristic search were obtained automatically by applying Neighbor-Join and BioNJ algorithms to a matrix of pairwise distances estimated using the JTT model and then selecting the topology with a superior log-likelihood value. A discrete Gamma distribution was used to model evolutionary rate differences among sites (5 categories (+G, parameter = 0.9002)). The rate variation model allowed for some sites to be evolutionarily invariable ([+I], 7.65% sites). This analysis involved 56 amino acid sequences. All positions containing gaps and missing data were eliminated (complete deletion option). There were a total of 2774 positions in the final dataset. The tree is drawn to scale, with branch lengths measured as the number of substitutions per site. Evolutionary analyses were conducted in MEGA11 [24]. The tree includes the polyproteins encoded in the genomes of the following species: agropyron mosaic virus (AgMV), Algerian watermelon mosaic virus (AWMV), banana bract mosaic virus (BBrMV), basella rugose mosaic virus (BaRMV), bean common mosaic necrosis virus (BCMNV), bean common mosaic virus (BCMV), beet mosaic virus (BtMV), blue squill virus A (BSVA), canna yellow streak virus (CaYSV), chilli ringspot virus (CRSV), catharanthus mosaic virus (CatMV), cocksfoot streak virus (CSV), cowpea aphid-borne mosaic virus (CABMV), cucurbit vein banding virus (CVBV), cyrtanthus elatus virus A (CEVA), dasheen mosaic virus (DsMV), East Asian passiflora virus (EAPV), freesia mosaic virus (FreMV), fritillary virus Y (FVY), gomphocarpus mosaic virus (GoMV), hardenbergia mosaic virus (HarMV), hippeastrum mosaic virus (HiMV), hordeum mosaic virus (HoMV), Iranian johnsongrass mosaic virus (IJGMV), iris severe mosaic virus (ISMV), johnsongrass mosaic virus (JGMV), keunjorong mosaic virus (KjMV), maize dwarf mosaic virus (MDMV), Moroccan watermelon mosaic virus (MWMV), narcissus degeneration virus (NDV), ornithogalum stripe mosaic virus (OrMV), onion yellow dwarf virus (OYDV), papaya leaf distortion mosaic virus (PLDMV), papaya ringspot virus (PRSV), passiflora chlorosis virus (PaCV), passionfruit Vietnam virus (PVNV), passion fruit woodiness virus (PWV), peanut mottle virus (PeMoV), pennisetum mosaic virus (PenMV), potato virus A (PVA), potato virus V (PVV), potato virus Y (PVY), ryegrass mosaic virus (RGMV), shallot yellow stripe virus (SYSV), sorghum mosaic virus (SrMV), soybean mosaic virus (SMV), sugarcane mosaic virus (SCMV), telosma mosaic virus (TelMV), watermelon mosaic virus (WMV), wisteria vein mosaic virus (WVMV), zantedeschia mild mosaic virus (ZaMMV), zucchini tigré mosaic virus (ZTMV) and zucchini yellow mosaic virus (ZYMV). The position of PaCV is highlighted in red and marked by an asterisk.

The latter genome sequence was 9672 bp long [excluding the poly(A) tail] and consisted of a 174 bp 5′ untranslated region (UTR), an open reading frame (ORF) encoding a 3084 aa polyprotein, and a 243 bp 3′ UTR (Figure 1A,B). In order to define the 5′ terminal sequence of the genome more precisely, we also performed 5′ RACE (rapid amplification of cDNA ends) experiments and sequenced the resulting amplification products. These experiments were compatible with two distinct genome termini, one closely matching the beginning of the assembled sequence and another at position +30 of the sequence deposited in GenBank. We conservatively deposited in GenBank the longest sequence. The 5′-UTR of this sequence had a characteristically low (5.17%) guanine content as compared to the ORF (23.73%) and the 3′-UTR (23.05%). The nucleotide sequence of CP was most similar (with identity levels ranging from 89.91 to 98.91%) to those of different isolates of PaCV [4]. These levels are above the currently accepted criteria for species demarcation in the *Potyviridae* family (i.e., 76–77% identity for the CP nucleotide sequence) [8,9], indicating that the assembled genome sequence belongs to a new isolate of the same species. We also prepared codon-based alignments of nucleotide sequences encoding the full-length polyproteins of representative species of the same genus (*Potyvirus*), and three species from a different genus (*Rymovirus*), which were used as the outgroup in the phylogenetic analysis. In BLAST searches, the nucleotide and amino acid sequences of the full-length polyprotein were most similar to those of BCMNV (NC_004047.1) [10], with overall identity levels (68.5% and 71.5%, respectively) below the corresponding thresholds for species demarcation (76% nucleotide identity and 82% amino acid identity). A maximum-likelihood phylogenetic analysis using these sequences clustered together the sequences of PaCV and BCMNV with 97% bootstrap support (Figure 2), showing that PaCV is a distinct species in the genus *Potyvirus*.

The polyprotein was predicted to be proteolytically cleaved into ten mature peptides (P1, HC-Pro, P3, 6K1, CI, 6K2, Nia-VPg, NIa-Pro, NIb, and CP) at nine conserved sites [11]: at the Y_317_/S dipeptide by the P1 proteinase, at the G_774_/G dipeptide by the helper component proteinase (HC-Pro), and at seven additional sites by the NIa-Pro proteinase (Figure 1A,C). Although some cleavage sites differed slightly from the most recent consensus sequences defined for NIa-Pro [12], they closely resembled the sites predicted for the polyproteins of bean common mosaic necrosis virus (BCMNV), bean common mosaic virus (BCMV) and cowpea aphid-borne mosaic virus (CABMV). In order to identify conserved sequence domains, the amino acid sequence of the polyprotein was analyzed using InterProScan version 5.51-85.0 in standalone mode, which allowed detecting hits to nine Pfam domains characteristic of potyviral proteins (Table 1). More specifically, these domains spanned known sequence motifs required for systemic movement and aphid transmission, including KLSC (located at amino acid positions 370–373), CCC (608–610), and PTK (626–628) motifs in HC-Pro, as well as a DAG motif (1689–1691) near the N-terminus of the coat protein (CP) [13,14,15]. In line with the presence of these motifs, PaCV has previously been reported to be transmitted by the green peach aphid (*Myzus persicae*) in *Bituminaria bituminosa* plants [7]. The sequence of HC-Pro also contains short motifs that have been linked to symptom expression and severity, such as FRNK (497–500) and CDNQLD (513–518) [16]. A conserved PIPO (“Pretty Interesting Potyvirus ORF”) sequence starts at a canonical GA_6_ motif (located at nucleotide positions 2950–2956), but only a single read (out of 53 reads spanning that region of the genome) contained an insertion (+A) supporting the existence of transcripts compatible with a P3N-PIPO trans-frame fusion protein [17,18]. The N-terminal region of the genome-linked VPg protein is rich in lysine and arginine residues, which are likely to be involved in its nuclear localization. Other conserved motifs, typical of potyviral proteins, were also identified in the amino acid sequences of CI (related to NTP binding and helicase activity) and NIb (related to its polymerase activity), as shown in Table 1.

To enable virus detection and quantification, we designed primers PaCV_F and PaCV_R for quantitative PCR (see Section 4). RNA was isolated in triplicate from leaf tissues of chlorotic and healthy-looking plants and reverse transcribed. After PCR amplification, the virus could only be detected in samples from plants exhibiting chlorosis symptoms (Figure 1D). The levels of the virus were normalized using the expression levels of the passion fruit PeCAC gene, which was amplified using primers PeCAC_F and PeCAC_R, and expressed relative to the sample with the lowest detected levels (those of a slightly chlorotic plant). We found that the virus levels were 34 times higher in chlorotic leaves than in leaves in which chlorosis was only slightly noticeable. Further research will be required to evaluate the specificity, efficiency, and sensitivity of these primers, as well as the stable expression of the plant gene used for normalization under our experimental conditions. The study of additional samples will help to assess how the levels of PaCV and PLV affect the observed foliar symptoms.

Recombination is known to have influenced the evolution of plant RNA viruses [18], leading to trees with incongruent topologies when the phylogeny of different parts of the viral genome is considered. To investigate the role of recombination during the evolution of the PaCV genome, we used the maximum likelihood (ML) method to construct unrooted phylogenetic trees separately for eight selected regions (P1, HC-Pro, P3, CI, VPg, NIa-Pro, Nib, and CP) of the polyproteins encoded in the genomes of PaCV and six additional potyviral species, including some closely related (BCMNV and CABMV) as well as other more distantly related species that might infect passion fruit (BCMV, TelMV, WMV and EAPV, the latter represented by two isolates in our analysis). The topologies of the trees that maximized the likelihood for each of these regions are shown in Supplemental Figure S1. The ML trees for P1, NIb, and HC-Pro all had a clade containing both PaCV and BCMNV, in agreement with the topology of the tree for the complete polyproteins (Figure 2). Like this tree, the ML trees for P3, CI, NIa-Pro and CP also contained a clade containing CABMV, PaCV, and BCMNV, albeit not necessarily in the same branching orders, which were sometimes supported by relatively low bootstrap values. The bootstrap consensus (BC) trees (Supplemental Figure S2), which capture the most frequent clusters present in the bootstrap replicates, were in general congruent with the topologies of the ML trees, although in some cases with differences in the branching order for CABMV, PaCV, and BCMN. As an example, the P1 sequence of PaCV was clustered with the sequence of BCMNV in the ML tree (as in 30% of the bootstrap replicates), but with that of CABMV in the BC tree (present in 42% of the replicates). The BC trees for Nib and HC-Pro each had a cluster formed by the sequences from CABMV and PaCV, and the trees for P1, P3, CI, NIa-Pro, and CP each had a cluster containing PaCV, CABMV, and BCMNV. The ML and BC trees for VPg had the sequences from TelMV and CABMV assigned to the same cluster with a bootstrap support of 90%. Similarly, the trees for NIb had the sequences for BCMV and CABMV assigned to the same cluster with 74% bootstrap support. These observations suggest that recombination might have influenced the evolution of VPg and NIb in these viruses. Indeed, recombination between BCMV and a different potyvirus, PVY, has been known for a long time [19]. However, we did not find convincing evidence supporting the possible involvement of recombination in the evolution of the PaCV genome beyond its inconsistent clustering with either BCMNV or CABMV in several trees, which might also be attributed to differences in the evolutionary rates (among the regions considered) or in the multiple sequence alignments (e.g., in the number of informative sites available in each of them). However, our recombination analyses would undoubtedly benefit from the availability of additional sequence data from PaCV and other species, which will allow us to define the role of recombination in the evolution of their genomes more reliably.

## 3. Conclusions

To our knowledge, the genome sequence described in this work is the first complete sequence deposited in GenBank for an isolate of PaCV. Although this species was first described in 2004, the species definition was based only on some partial sequences, mainly from the region encoding the coat protein. The availability of this complete sequence has allowed us to gain insight into the genome structure and phylogenetic relationships of this virus, which demonstrate that PaCV is correctly classified as a distinct species in the genus *Potyvirus*. Using the genome sequence, we designed oligonucleotides that have allowed us to detect the virus in samples from different tissues of infected passion fruit plants using quantitative RT-PCR. 

## 4. Materials and Methods

### 4.1. Plant Material

For the transcriptome assembly, we collected ovary tissues of passion fruit (*Passiflora edulis* Sims) plants grown in 2018 in the experimental farm of the Faculty of Agriculture of the Hebrew University of Jerusalem at Rehovot, Israel. For the detection and quantification of the virus, additional leaf samples were collected from representative plants exhibiting symptoms of virus infection, as well as from non-symptomatic plants, which were used as the negative control. 

### 4.2. Genome Assembly and Annotation

Total RNA was extracted using the CTAB method. Sequencing libraries were prepared with the TruSeq RNA Library Preparation Kit v2 (Illumina, San Diego, CA, USA) and sequenced using an Illumina NextSeq 500 sequencer. The resulting single-end, unstranded 76 bp reads were assembled by running Trinity version 2.4.0 [20] in unpaired read mode. To correct the assembled sequence, the reads were iteratively mapped back to the assembled genome with Bowtie2 version 2.3.4 using default parameters [21]. Sequence differences between the reads and the assembly were detected with the mpileup command of Samtools version 1.3.1 and the call -cv command of Bcftools version 1.3.1 and subsequently used to modify the genome sequence as required [22]. 

### 4.3. 5′ Rapid Amplification of cDNA Ends (5′-RACE)

Reverse transcription coupled with template switching (RT-TS) reactions were performed using 1 μL of RNA of two different biological samples with the Template Switching RT Enzyme Mix from New England Biolabs (NEB M0466). Primer annealing was performed with either PaCV567 (5′-CAGTCTTTTGCGTGCATTAGCTTC-3′) or PaCV588 (5′-CTCTTTTTCACGCAGTTCCTTCAG-3′) as reverse primers, followed by a reverse-transcription and template-switching step involving the TSO-RACE primer (5′-GCTAATCATTGCAAGCAGTGGTATCAACGCAGAGTACATGGG-3′) in a 10-μL reaction. Prior to their use as a template, 10 μL of water was added to each reaction. One μL of this RT-TS reaction was used as a template for PCR involving the TSO-PCR primer (5′-CATTGCAAGCAGTGGTATCAAC-3′) combined either with VSP2 (5′-ACTCTTAAGGGAGGGTCGCT-3′) or VSP3 (5′-CAAGCTTGATGCGACTCACAC-3′) primers at 60 °C of hybridization temperature. Nested-PCR were performed afterward using 1 μL of a 1:100 dilution of the previous PCR products as a template. The PCR reactions with VSP2 were used as a template for nested-PCR using VSP4 (5′-GGCTTTGGCTCTCCGTTG-3′) and TSO-PCR primers, and PCR reactions with VSP3 were used as a template for nested-PCR using combinations of VSP1 (5′-ATATCCATGGCCTTAACTCCAGC-3′) or VSP2 and TSO-PCR primers. Selected PCR products were sequenced by StabVida from both ends using TSO-PCR and the appropriate reverse primer (VSP1, VSP2, or VSP4).

### 4.4. Sequence Alignment and Phylogenetic Analysis

Multiple sequence alignments were performed and refined with MUSCLE v. 3.8.31 [25]. MEGA 11 was used to build phylogenetic trees using the maximum likelihood method [24]. 

### 4.5. Detection of PaCV Using Quantitative RT-PCR

Separate RNA extractions were performed in triplicate for samples of leaf tissue from asymptomatic, slightly chlorotic, and clearly chlorotic plants. After reverse transcription, cDNA samples were amplified using qPCRBIO SyGreen Mix Hi-ROX (PCR Biosystems Ltd., London, UK). Reactions were run on a qTOWER^3^ real-time PCR thermal cycler (Analytik Jena AG. Jena, Germany). Primers used for PaCV detection were PaCV_F (5′-GATACTCAGCCTTGATCACCTCA-3′) and PaCV_R (5′-CGCACCAGACCATA-AAGCCA-3′). The housekeeping PeCAC gene (GenBank KY471459) was amplified with the PeCAC_F (5′-TCAAGAGGGAGTGCGTTCAC-3′) and PeCAC_R (5′-CAACCAACAGCGCCTGTAAC-3′) primers and used as an endogenous control [26]. A standard curve was obtained for each amplicon using a fragment containing its amplified region. Quantification of each amplicon was performed using qPCRsoft 3.4, qTOWER^3^ Analytic Jena software. Relative levels of PaCV in each sample were calculated by dividing the PaCV level by the PeCAC level (both in arbitrary units), and finally normalized by setting the value of the sample with the lowest detected level at 1.

## Figures and Tables

**Table 1:** Results of an InterProScan search for conserved Pfam domains present in the polyprotein of PaCV.

Pfam	Name	Location (aa)	Peptides	E-Value
PF01577	Potyvirus P1 protease	79–316	P1	2.1 × 10^−43^
PF00851	Helper component proteinase	341–774	HC-Pro	3.2 × 10^−111^
PF13608	Protein P3 of Potyviral polyprotein	787–1224	P3, 6K1, CI	5.7 × 10^−57^
PF07652	Flavivirus DEAD domain	1272–1387	CI	6.8 × 10^−7^
PF00271	Helicase conserved C-terminal domain	1418–1532	CI	4.2 × 10^−10^
PF08440	Potyviridae polyprotein	1558–1828	CI, 6K2	9.0 × 10^−63^
PF00863	Peptidase family C4	2050–2282	NIa-Pro	2.5 × 10^−62^
PF00680	Viral RNA-dependent RNA polymerase	2340–2751	NIb	8.8 × 10^−74^
PF00767	Potyvirus coat protein	2850–3080	CP	1.2 × 10^−87^

## Data Availability

The genome sequence has been deposited in the GenBank database with accession number MT263075. The raw reads for the genome assembly have been submitted to the BioProject database with accession number PRJNA853795.

## Supplementary Materials

The following supporting information can be downloaded at: https://www.mdpi.com/article/10.3390/plants11141838/s1, Supplemental Figure S1: Maximum likelihood phylogenetic trees for eight different regions of the polyprotein. Supplemental Figure S2: Bootstrap consensus trees for the ML phylogenies obtained for eight different regions of the polyprotein. In both figures, the internal branches are labeled with the bootstrap percentages.
