# Supplementary figures and images for: Complete Genome Sequence of an Isolate of Passiflora chlorosis virus from Passion Fruit (Passiflora edulis Sims)
Source: Plants (Basel). 2022 Jul 13;11(14):1838. doi: 10.3390/plants11141838 (PMC9317278; doi:10.3390/plants11141838)

Supplemental Figure 1

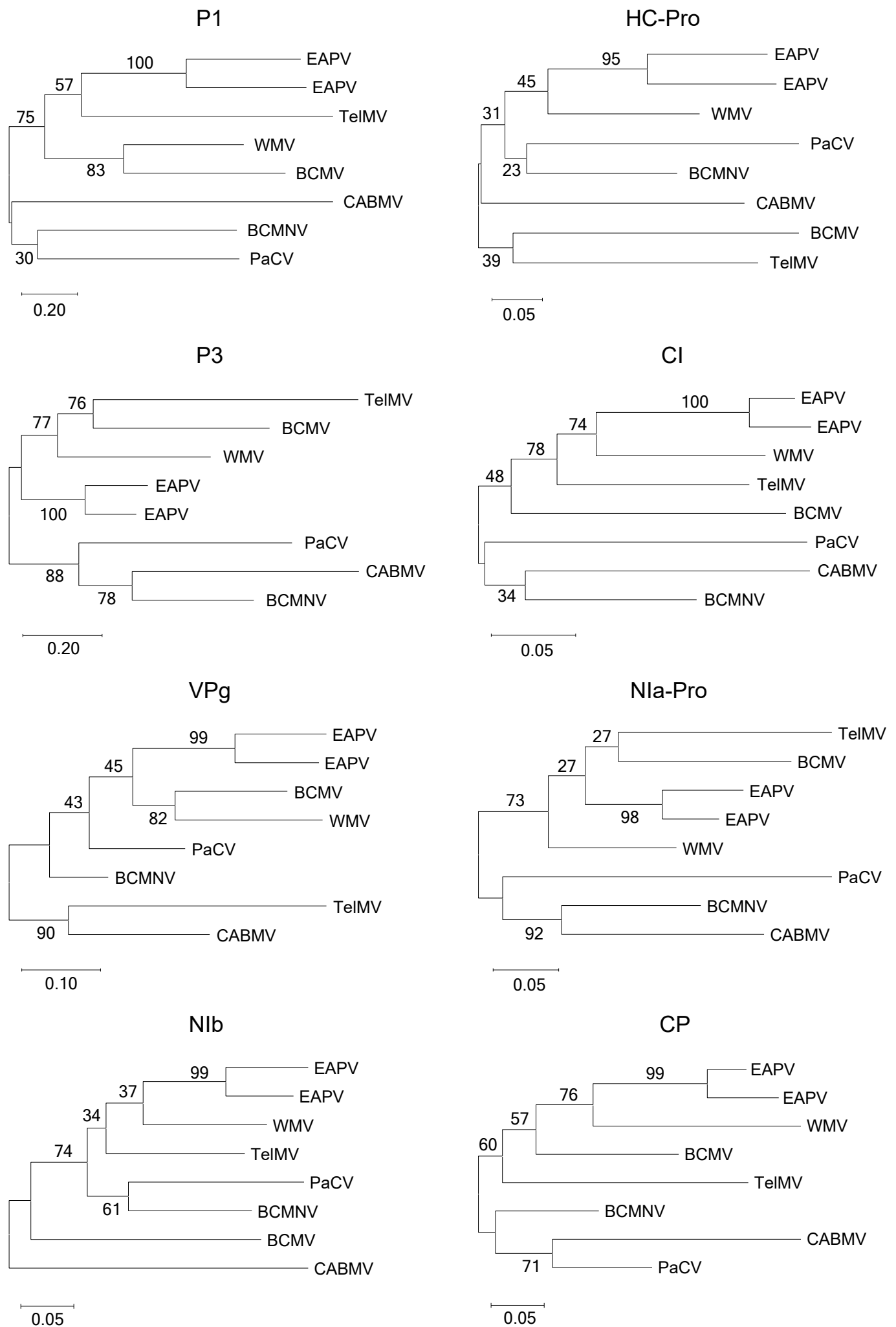

Supplemental Figure 2

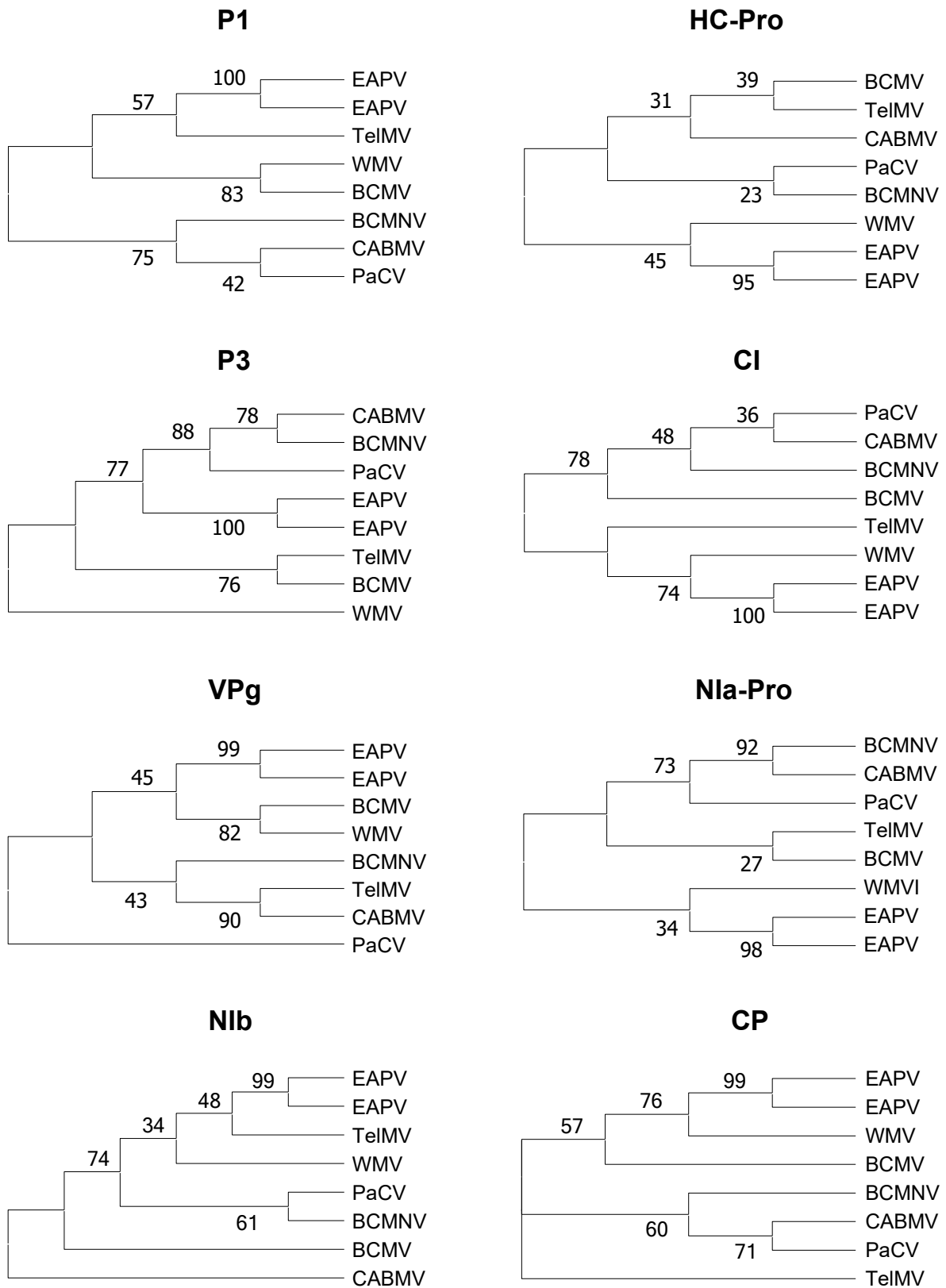

Supplement: Supplementary file 1 [file plants-11-01838-s001.zip › plants-1767739-supplementary.pdf]
